# Communicative-Pragmatic Treatment in Schizophrenia: A Pilot Study

**DOI:** 10.3389/fpsyg.2016.00166

**Published:** 2016-02-23

**Authors:** Francesca M. Bosco, Ilaria Gabbatore, Luigi Gastaldo, Katiuscia Sacco

**Affiliations:** ^1^Department of Psychology, Center for Cognitive Science, University of TurinTurin, Italy; ^2^Neuroscience Institute of TurinTurin, Italy; ^3^Faculty of Humanities, Research Unit of Logopedics, Child Language Research Center, University of OuluOulu, Finland; ^4^AslTo2 Department of Mental HealthTurin, Italy; ^5^Brain Imaging GroupTurin, Italy

**Keywords:** rehabilitation, schizophrenia, pragmatic, communication, training

## Abstract

This paper aims to verify the efficacy of Cognitive Pragmatic Treatment (CPT), a new remediation training for the improvement of the communicative-pragmatic abilities, in patients with schizophrenia. The CPT program is made up of 20 group sessions, focused on a number of communication modalities, i.e., linguistic, extralinguistic and paralinguistic, theory of mind (ToM) and other cognitive functions able to play a role on the communicative performance, such as awareness and planning. A group of 17 patients with schizophrenia took part in the training program. They were evaluated before and after training, through the equivalent forms of the Assessment Battery for Communication (ABaCo), a tool for testing, both in comprehension and in production, a wide range of pragmatic phenomena such as direct and indirect speech acts, irony and deceit, and a series of neuropsychological and ToM tests. The results showed a significant improvement in patients’ performance on both production and comprehension tasks following the program, and in all the communication modalities evaluated through the ABaCo, i.e., linguistic, extralinguistic, paralinguistic, and social appropriateness. This improvement persisted after 3 months from the end of the training program, as shown by the follow-up tests. These preliminary findings provide evidence of the efficacy of the CPT program in improving communicative-pragmatic abilities in schizophrenic individuals.

## Introduction

People with schizophrenia experience symptoms such as delusions, hallucinations, disorganized speech and behavior, that cause difficulty in social relationships (DSM 5; American Psychiatric Association [APA], 2013). In the clinical pragmatic domain (Cummings, 2014), the area of study of pragmatic impairment in patients with communicative disorders, several studies have reported that communicative ability is impaired in patients with schizophrenia (Langdon et al., 2002; Bazin et al., 2005; Linscott, 2005; Marini et al., 2008; Colle et al., 2013). For example, Bazin et al. (2005), created a structured interview, the Schizophrenia Communication Disorder Scale, which they administered to patients with schizophrenia. The authors observed that these patients performed less well than those affected by mania or depression in managing a conversation on everyday topics, such as family, job, hobbies, and so on. Likewise, non-compliance with conversational rules, such as consistency with the agreed purpose of the interaction, giving the partner too little or too much information and failing to be clear and concise, have been observed to a greater extent in communicative interactions between people with schizophrenia, than in those involving healthy controls; furthermore, such patients have difficulty in using non-verbal cues to facilitate the communicative partner’s engagement (Linscott, 2005). In line with such study, Marini et al. (2008) observed that patients with schizophrenia have impaired narrative skills.

Moreover, focusing on specific communicative-pragmatic phenomena, studies in the literature have observed impairments in people with schizophrenia, when compared with healthy controls, in adhering to Grice’s maxims, i.e., when a person says something that is not coherent, or not true or not adequate with respect to the context (Tényi et al., 2002; Mazza et al., 2008), in recognizing and repairing communicative failures (Bosco et al., 2012b), in the comprehension of indirect speech acts (Corcoran, 2003), deceitful statements (Frith and Corcoran, 1996), and ironic and other figurative expressions, i.e., metaphors and idioms (Langdon et al., 2002; Tavano et al., 2008).

Prosody and facial expression recognition, abilities that are necessary in order to comprehend emotions in everyday communicative interactions, are also impaired in individuals with schizophrenia (for a review, see Edwards et al., 2002).

Colle et al. (2013) recently provided a broad description of communicative abilities in patients with schizophrenia, using the Assessment Battery for Communication (ABaCo; Sacco et al., 2008; Angeleri et al., 2012; Bosco et al., 2012a). The authors showed that patients with schizophrenia performed less well, when compared to healthy controls, both in the comprehension and in the production of several kinds of pragmatic phenomena, such as indirect speech acts, deceitful and ironic utterances, and had difficulty in using different expressive modalities, i.e., linguistic, extralinguistic, and paralinguistic.

Although the relevant literature on this topic agrees in recognizing that patients with schizophrenia have impaired communicative-pragmatic abilities, and difficulties with conveying meaning using language, extralinguistic, i.e., non-verbal, and paralinguistic cues, to our knowledge no specific rehabilitation program focused specifically on such problems has yet been developed in order to help patients to overcome their difficulties in this domain.

Beside their impairment in communicative-pragmatic skills, patients with schizophrenia exhibit a deficit (e.g., Frith, 2004; Bosco et al., 2009; Brüne et al., 2011) in theory of mind (ToM), i.e., the capacity to attribute mental states to oneself and to others, and to use such knowledge to interpret one’s own and other people’s behaviors (Premack and Woodruff, 1978). Labels that refer to similar, albeit broader abilities are for example metacognition (Flavell, 1979) and social cognition (Adolphs, 2003). Frith (1992) was the first author to explain the communicative-pragmatic impairment of individuals with schizophrenia on the basis of their principal deficit in ToM. The author proposed that in a communicative interaction patients with schizophrenia fail to correctly take into account the partner’s mental states, for example intention, desire and belief, and that this deficit can make their discourse bizarre, unintelligible and obscure. Patients with schizophrenia may fail to correctly interpret a partner’s mental states because they either under-attribute mental states, i.e., they are not able to detect the other person’s communicative intentions, or they over-attribute mental states, for example they attribute a communicative intention to a person who has absolutely no desire to communicate with them (see also Abu-Akel and Bailey, 2000).

In line with such empirical evidence, some rehabilitation treatment programs, specifically focused on impaired ToM, social and metacognitive abilities, have been developed in order to improve such competences in patients with schizophrenia (Roncone et al., 2004; Moritz et al., 2005, 2010; Kayser et al., 2006).

However, if on one side the capacity to mind-read needs to be intact in order to comprehend a partner’s communicative intention (Happé and Loth, 2002; Salvatore et al., 2008), on the other side, several authors have agreed that communicative-pragmatic competence cannot be entirely and exclusively identified with the ability to mind-read (Sperber and Wilson, 2002; Tirassa et al., 2006a,b; Tirassa and Bosco, 2008). In a recent study (Bosco et al., 2012b) showed that ToM is only partially able to explain the difficulty that individuals with schizophrenia have in recognizing and repairing a communicative failure.

The present research sets out to provide preliminary empirical evidence concerning the efficacy of a recently developed rehabilitation intervention, Cognitive Pragmatic Treatment (CPT), in a group of patients with schizophrenia. The CPT was originally developed to recover pragmatic abilities in patients affected by neuropsychological disorders following brain injury, i.e., traumatic brain injury (TBI), as well as those with psychiatric disorders, i.e., schizophrenia. Despite differences in the etiology and the clinical profile of these pathologies, patients with acquired brain injury and schizophrenia encounter similar communicative difficulties. For example they share an impaired ability to go beyond the literal meaning of utterances, and thus to correctly interpret indirect speech acts, metaphors, and irony (see Angeleri et al., 2008); moreover, these patients have difficulties in producing requests and exhibit a deficit in integrating information, with low levels of adherence to the context (see Cummings, 2014). CPT has already shown to be effective in improving and enhancing communicative-pragmatic abilities in TBI patients (Gabbatore et al., 2015).

The CPT program was developed within the pragmatic domain (Austin, 1962; Searle, 1979; Grice, 1989); from this theoretical perspective, human communication is a form of cooperative social interaction between people who want to share some of their knowledge with one or more individuals (Grice, 1989). The assumption underlying this area of study is that in communicative interactions there is often a gap between what is literally said, and what the speaker actually wants to communicate. For example, a person could say “What a beautiful blouse you’re wearing” with the intention of being sincere, ironic or misleading, depending on a specific context/situation. From this perspective, it is not possible to establish a univocal correspondence between a sentence and its communicative meaning, and pragmatics deals with the communicative meaning that a particular utterance can assume in the context within which it is pronounced. Several components have to be taken into consideration in order to explain the complexity of human communication: the knowledge shared by the participants in a dialog at a given time (Clark, 1992), the speaker’s communicative intention in proffering the speech act and the inferential processes allowing the interlocutors to comprehend the speaker’s intended meaning starting from the literal one (Searle, 1979; Grice, 1989).

More specifically, the CPT program was developed on the basis of the CPT (for the most recent developments in this theory see Bara, 2010), which focuses on the inferential processes underlying human communication (see also Bosco et al., 2004, 2006, 2013, 2015; Bosco and Bucciarelli, 2008 for a full description of the theoretical framework). According to the theory, a communicative act can be conveyed through different modalities – words, gestures, body movements, and facial expressions – which should be considered as different means to express a particular communicative meaning. One of the relevant aspects of the theory is that communication is conceived as an inferential process through which the partner is able to comprehend the speaker’s intended meaning starting from the literal meaning of the utterance (for details see Airenti et al., 1993; Bara, 2010). In particular, CPT is focused on the following communication modalities: linguistic, extralinguistic, – i.e., non-verbal – and paralinguistic, – i.e., rate, pitch and volume of voice, prosodic cues, such as rhythm and intonation. The training program is also focused on social appropriateness, meaning a person’s sensitivity to the social context such as, for example, the capacity to reply politely to a question put kindly; finally, CPT is focused on conversational ability, i.e., the ability to manage turn-taking and the topic of conversation.

As a final point, some authors have proposed that an impairment of cognitive functions such as, for example attention, memory and planning, could be considered the core feature of schizophrenia, and that such impairment could be regarded as primary with respect to others (see Reichenberg and Harvey, 2007). Furthermore, a study by Sponheim et al. (2003) found a correlation between impairment in planning and working memory and patients’ difficulty to solve a pragmatic task, i.e., proverb comprehension. For exploratory purposes we thus also administered a battery of neuropsychological tests, in addition to ToM tasks, in order to verify whether the improvement we expected to observe in patients’ communicative-pragmatic ability was specific to this ability or also detectable in other ones, i.e., ToM, learning and memory.

In summary, we hypothesized that the CPT program would be able to improve the communicative-pragmatic skills of patients with schizophrenia in all of the communication modalities dealt with in the program. Moreover, we expected such improvement to persist after a follow-up period of 3 months. For exploratory purposes we also investigated whether the effects of our training program were specific to communicative abilities or also regarded other cognitive abilities, i.e., planning, memory (working memory and long term memory), and learning.

## Materials and Methods

### Participants

Twenty-three patients with a diagnosis of schizophrenia according to the DSM-IV (American Psychiatric Association [APA], 2000) were recruited for this study. Six of the patients did not complete the rehabilitation training owing to personal and health problems encountered at the time of the study (e.g., they moved to another local health district within the city, or were included in a supported employment program). Thus, the results of the study refer to a sample of 17 patients with schizophrenia (see **Table 1** for a detailed description of the sample). A diagnosis of schizophrenia was assigned by qualified clinicians working at the clinical units, using DSM-IV criteria.

**Table 1:** Clinical details of participants (*N* = 17).

	Participants ID																
	1	2	3	4	5	6	7	8	9	10	11	12	13	14	15	16	17
Sex	F	F	M	M	M	F	F	F	M	M	M	M	M	F	F	M	M
Age	34	39	39	29	40	44	48	41	51	32	45	41	42	61	32	49	42
Education (years)	8	8	13	17	8	13	8	8	8	13	13	13	8	13	18	13	8
Illness duration (years)	15	6	3	10	10	19	20	5	30	7	18	15	21	30	2	19	22
PANNS												
Negative symptoms	15	7	15	32	13	–	21	10	8	7	9	22	26	21	9	11	8
Positive symptoms	30	25	15	30	15	–	28	25	34	22	11	31	37	19	26	31	34
General symptoms	67	45	36	60	31	–	60	43	25	27	33	56	55	41	45	43	31
Total score	112	77	*66*	122	59	–	109	78	67	56	53	109	118	81	80	85	73
MMSE	24.75	29.42	30	27.07	29.62	29.9	29.9	28.62	26.97	30	25.9	28.89	24.62	28.49	25.10	27.89	26.62

The clinical sample was made up of 7 females and 10 males, aged 29–61 (*M* = 41.65 years; *DS* = 7.84) and with 8–18 years of formal education (*M* = 11.18 years; *DS* = 3.24). All the participants were outpatients of the Turin district health authority and were recruited through the collaboration with the not-for-profit association Di.A.Psi and the AslTo2 Department of Mental Health in Turin. All patients were chronically ill with a disease onset of between 2 and 30 years prior to recruitment in the study (*M* = 15.27; *DS* = 8.61). The patients experienced different degrees of autonomy: four were able to live independently or with minimal support, seven lived with their families and the other six lived in sheltered accommodation or at rehabilitation units. None required chronic hospitalization at the time of the study.

At the time of the first assessment, the patients’ symptomatology was investigated by qualified psychiatrists using the Positive and Negative Syndrome Scale (PANSS; Kay et al., 1987). This scale consists of thirty items divided into three scales: the first scale assesses positive symptoms (seven items), the second one negative symptoms (seven items) and the third represents a general psychopathology scale (16 items); each item is evaluated on a seven-point Likert scale ranging from *absent* (1) to *extremely severe* (7). The participants’ PANSS scores are shown in **Table 1**. Three of the patients were taking typical antipsychotic medications and thirteen were on atypical antipsychotics; seven of the patients were also being treated with other medications, including antiepileptic, anxiolytic, and cardiac medications.

Inclusion criteria for the study were: (1) at least 18 years of age; (2) no acute or florid psychotic state, all patients were tested in their chronic phase; (3) Italian native speakers; (4) in possession of adequate cognitive skills, tested by the achievement of a cut-off score on the Mini Mental State Examination (MMSE; Folstein et al., 1975; cut-off >24/30); (5) communicative-pragmatic impairment, as resulting from the administration of form A of the Assessment Battery for Communication (Sacco et al., 2008; Bosco et al., 2012a) in comparison to normative performance on the ABaCo (Angeleri et al., 2012) by healthy individuals. Finally, (6) a minimum attendance rate of 60% at all therapy sessions was mandatory for inclusion in the present study.

Exclusion criteria were (1) leucotomy, (2) neurological disability (3) alcohol or drug addiction, evaluated on the basis of anamnestic data from the case history of each patient. All the participants gave their written informed consent to participate in the research. Approval for the study had previously been obtained from the Bio-ethics committees of both the University and the AslTo2 Department of Mental Health of Turin.

### Experimental Design

**FIGURE 1:**
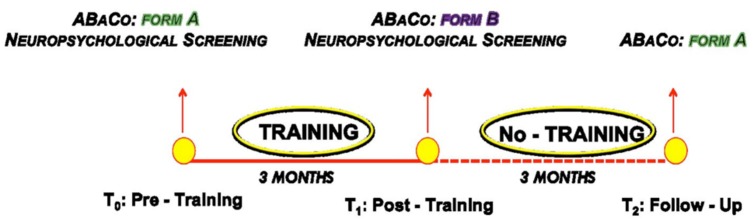
**Graphical representation of the experimental design**.

The 6-months study period comprised a 3-months training period and three experimental sessions, using an ABA design (see **Figure 1**).

#### T0_Pre-Training

In order to have a measure of the patients’ abilities before embarking on the rehabilitation program, their communicative skills were assessed the week before the treatment started, using Form A of the ABaCo. A neuropsychological and ToM test battery was also administered to the patients pre- and post-treatment, to ascertain whether the expected improvement could be generalized for other cognitive abilities or was specific to their communicative-pragmatic abilities (see **Table 2**).

**Table 2:** Neuropsychological and theory of mind tests.

Domain	Neuropsychological test	Description
Selective attention	Attentive Matrices (Italian standardized version in Spinnler and Tognoni, 1987)	Patterns of numbers are displayed on a sheet and the patient is required to find the target digits among non-relevant ones. The tasks are presented according to a trend of increasing complexity (1–3 digits to be found) and scores are attributed according to both accuracy and completion time
Divided attention	Trail Making test (Reitan, 1958)	The test is structured in two parts (A and B), both consisting of 25 circles arranged on a sheet of paper. Part A: the patient is required to draw lines to connect the circles (1–25) in ascending order. Part B: the circles contain both numbers (1–13) and letters (A–L) and the patient is required to connect the circles in ascending order, in an alternating sequence of numbers and letters (i.e., 1-A-2-B, etc.). The patient is asked to complete the task as quickly as possible. Direct scores for part A and part B are assigned according to the completion time. Specifically, we used the B–A difference score, commonly used in clinical settings as a pure indicator of executive control abilities.
Verbal short-term memory	Verbal Span (Italian standardized version in Spinnler and Tognoni, 1987)	The patient is required to repeat sequences of words straight after the examiner. Each word is made up of two syllables, and the level of complexity of the sequences increases progressively, ranging in length from 1 to 9 words. Scores are attributed according to the longest series in which two or more sequences are correctly repeated.
Spatial short-term memory	Spatial Span (Italian standardized version in Spinnler and Tognoni, 1987)	Nine wooden blocks are arranged irregularly on a wooden panel. The examiner taps the blocks in random sequences of increasing length. The patient is asked to repeat the sequence, tapping the blocks himself immediately after the examiner. The length of the tapping sequences increases progressively (from 2 to 10 blocks). Scores are attributed according to the length of the sequence in which the patient repeats at least two taps correctly.
Verbal long-term memory	Immediate and Deferred Recall test for long-term verbal memory (Italian standardized version Spinnler and Tognoni, 1987)	A short story is read aloud by the examiner and the patient is immediately required to freely recall it. After the first recall, the examiner reads the story again. Ten minutes later (after carrying out a non-verbal interfering activity), the patient is required to recall the details of the story once again (deferred recall). A score is attributed to both the immediate and the deferred recall, based on how many relevant elements of the story are mentioned.
Planning ability	Tower of London (Shallice, 1982)	This is a problem-solving task, requiring the patient to rearrange three colored rings, starting from their initial position on three upright sticks, to a new set of predetermined positions. The patient is asked to reach the goal-rearrangement in as few moves as possible and in accordance with simple given rules (e.g., do not move more than 1 ring at a time). Scores are attributed according to accuracy and completion time.
Cognitive flexibility	Modified Card Sorting test (MCST; Nelson, 1976)	The test material consists of four stimulus cards and a number of response cards containing several symbols (different in color, number, and type of shape). The patient is asked to complete a sorting process, placing each response card below one of the stimulus cards. Each response card has just one feature in common with three of the stimulus cards, and none with the fourth one. The patient is not told what criterion (i.e., shape or color or number) he is supposed to use each time, but he/she is guided by the examiner to discover the sorting rule. Scores are attributed according to the number of categories completed and the number of errors.
Logical reasoning	Raven’s Standard Progressive Matrices (Raven, 1938)	This test is based on visual pattern matching and analogy problems pictured in non-representational designs. The patient is required to conceptualize spatial, design and numerical relationships of increasing difficulty, and to select the correct one in a multiple-choice design. The patient is shown the patterns with a set of incomplete figures and must complete the set choosing 1 of the 6 responses given below each pattern.
Linguistic ability	Aachener Aphasie test (AAT) denomination scale (Huber et al., 1983)	In the AAT-Denomination scale, the patient is required to name 40 items of increasing complexity, presented as images. The score is attributed on the basis of the accuracy of the answer.
Theory of mind	Sally and Ann task (Baron-Cohen et al., 1985)	This task involves the use of two paper dolls (Sally and Ann) acting in a false belief scenario. The patient is required to correctly interpret the characters’ behavior focusing on the beliefs attributed to them.
Theory of mind	Strange Stories task (Happé, 1994)	The task consists of a set of mentalistic stories (e.g., double bluff, mistakes, white lies). The patient is required to listen carefully to each story and answer some questions requiring an inference about the characters’ thoughts, feelings and intentions. Each story is scored separately and the total score is attributed by summing the scores obtained on each story. No time limit is given.

*Adapted from Gabbatore et al. (2015)*.

#### T1_Post-Training

A week after completing the training program, we used Form B of the ABaCo to assess the efficacy of the training program on the patients’ communicative abilities. We also evaluated their cognitive performance in the post-training phase by administering the same neuropsychological and ToM tests used at T0.

#### T2_FollowUp

We assessed the stability of patients’ communicative abilities again 3 months after the rehabilitation program, using Form A of the ABaCo.

### Training: Structure and Procedure

The Cognitive-Pragmatic Treatment program consisted of 20 sessions, each dealing with one particular aspect of communication. Patients attended two sessions a week, for 10 weeks. Each session lasted approximately one and a half hours, with a 10-min break. Patients attended the sessions in small groups of five/six, led by a psychologist (see **Table 3** for an overview of each session). The therapy mainly concentrated on the different expressive modalities of communication, i.e., linguistic, extralinguistic, paralinguistic, social appropriateness, and conversational abilities.

**Table 3:** Schematic structure of the Cognitive Pragmatic Treatment, reporting the topic, and the clinical tools of each session.

Week	Sessions order	Topic	Tools and procedures
1	1	Awareness of the deficit	Construction of the clinical setting and introduction of aims and tools of the CPT; Videorecording of the self-presentation of each patients (own communication difficulties and expectations).
	2	General communicative ability	Video-taped scenes and role playing focused on the overall pragmatic effectiveness expressed through all the modalities constituting communicative competence.
2	3	Linguistic ability	Video-taped scenes and role playing based on the linguistic expressive modality.
	4	Linguistic ability	Video-taped scenes and role playing based on the linguistic expressive modality.
3	5	Extra-linguistic ability	Video-taped scenes and role playing based on the gestural modality.
	6	Extra-linguistic ability	Video-taped scenes and role playing based on the gestural modality.
4	7	Paralinguistic ability	Video-taped scenes, facial expression recognition, and tone of the voice tasks, role playing; *Picture of Facial Affect (POFA*; Ekman and Friesen, 1976), and *JACfee and JACneuf* (Matsumoto and Ekman, 1988).
	8	Paralinguistic ability	Video-taped scenes, facial expression recognition, and tone of the voice tasks, role playing. *JACbart* (Matsumoto et al., 2000), and *Eyes Task-Adult* (Baron-Cohen et al., 1997)
5	9	Paralinguistic ability	Video-taped scenes, Facial expression recognition and tone of the voice tasks, role playing; *Cohn-kanade Database* (FACS model; Kanade et al., 2000), grammelot.
	10	Social appropriateness ability	Video-taped scenes and role playing focused on social appropriateness and communicative adequacy in different contexts.
6	11	Social appropriateness ability	Video-taped scenes and role playing focused on social appropriateness and communicative adequacy in different contexts.
	12	Conversational ability	Video-taped scenes, role playing and Tangram exercises focused on the use of conversational rules (i.e., turn-taking and management of the topic).
7	13	Conversational ability	Video-taped scenes, role playing and Tangram exercises focused on the use of conversational rules (i.e., turn-taking and management of the topic).
	14	Management of telephonic conversation	Audio-taped telephone conversations and role playing specifically focused on telephone conversational rules (i.e., no possibility to take advantage of the paralinguistic and gestural elements which usually connote communicative interactions).
8	15	Planning ability	Sub-goal task activities, both alone and in groups (e.g., planning household chores).
	16	Theory of mind	Video-taped scenes and role playing focused on the ability to formulate meta-representations with respect to one’s own and others’ mental states.
9	17	Theory of mind	Video-taped scenes and role playing focused on the ability to formulate meta-representations with respect to one’s own and others’ mental states.
	18	Narrative ability	Description tasks (Brookshire and Nicholas, 1997) and speech elicitation pictures (WAB; Kertesz, 1982) able to train the ability to tell a story or describe a situation, giving the right amount of information in the appropriate way.
10	19	General communicative ability	Video-taped scenes and role playing focused on the overall pragmatic effectiveness expressed through all the modalities constituting communicative competence.
	20	Post-training awareness	Conclusions and feedback about progresses made, compared to the initial video-recorded performance of each patient.

Some rehabilitation sessions also addressed other aspects of communicative ability such as awareness, ToM, and planning. The sessions provided an ecological setting where patients were encouraged to put their communicative abilities into practice and taught how to deal with the problems they encountered in daily communication, through self-monitoring strategies and feedback provided by the therapist. The various training activities centered on the idea that the ability to create new meanings and share them with other people, using different expressive modalities, i.e., linguistic, extralinguistic and paralinguistic, is the very essence of human communication (Bara, 2010). The goal was to help patients to interpret the intended meaning and to look beyond the literal one. Often in everyday communicative interactions the intended meaning does not simply correspond to the literal one, for example a person could say “It’s a really interesting book,” meaning to be ironic and remarking on the fact that it is boring and useless. Communication may be regarded as a process involving different elaboration stages, through which the individual is able to comprehend the partner’s intended communicative meaning, starting from the literal meaning of the actual sentence. The training program involved activities designed to improve patients’ inferential abilities so as to fill the gap that may exist between what is said and what is meant. The discussions and exercises proposed in each session focused on the communicative intentions observed rather than on the mere linguistic aspects of the utterances, which are fairly well-preserved in these patients. More specifically, patients were encouraged to go beyond the literal meaning and focus on the speaker’s communicative intentions and the possible alternative meanings and implications, depending on the circumstances.

The training program also focused on the ability to take contextual information into consideration, and modulate speech according to a particular context: schizophrenia often implies difficulties in decoding the violations of conversational implicatures and these patients often exhibit low levels of adherence to the context, so that their discourse is characterized by derailments and digressions. The communicative inappropriateness shown by subjects with schizophrenia is indeed a severe obstacle to their social reintegration. During the CPT program, particular emphasis was given to the ability to identify the other person’s intentions, without over-interpreting their mental states and thus jumping to wrong conclusions.

Each session was video-recorded, with the participants’ consent, and the video feedback was used during and at the end of the program. This allowed the experimenters to give a better analytical, critical, and objective contribution to the contents of the sessions and helped to make patients more aware of their impairment and of the progress they had made. The general structure of each rehabilitation session is described in Appendix A (Sheet 1 – Supplementary Material), where some examples of the rehabilitation tools and exercises used during the training program are also provided.

### Measures

We used the equivalent forms (A and B) of the Assessment Battery for Communication (Bosco et al., 2012a) to evaluate the effects of the treatment. Equivalent forms of the same test are useful in clinical practice and intervention research, for testing patients’ performance at different times, pre- and post-rehabilitation. Such forms envisage the use of test and retest procedures to measure the effectiveness of the treatment; they also reduce the possibility of practice and memory affecting patients’ scores when being retested, instead of these representing a real measure of their progress. The equivalent forms of the ABaCo consist of four different evaluation scales – linguistic, extralinguistic, paralinguistic, and context – which assess all the main pragmatic aspects of communication. Each scale is, in turn, divided into a *comprehension* and a *production* subscale evaluating the respective abilities in each communication modality.

A series of neuropsychological and ToM tests were administered before (T0) and after the training program (T1; see **Table 2**).

### Coding Procedures

Participants’ answers on the ABaCo were coded off-line and their scores were recorded on specific score sheets while watching the video-recorded sessions.

The rater who evaluated the patients’ performance did not take part in administering the battery and was blind to the aims of the study. Performance was rated on various dimensions, derived from the CPT. These dimensions may be regarded as the steps to be taken in order to understand or produce the relevant communicative phenomena: the more complex the pragmatic phenomena, the more steps they involve. On the linguistic and extralinguistic scales, dimensions are represented by the comprehension of (a) the literal message, (b) the meaning and implication of the utterance/gesture and, in the most complex communicative acts, (c) the aim (e.g., to deceive or to be ironic). Patients scored 1 for each item in which they passed on all dimensions, and 0 for each item in which they did not pass on all dimensions. As far as production tasks are concerned, 1 mark is obtained for the production of a communicative act (utterance or gesture, respectively) that is (a) congruent with respect to the question and (b) fulfills the requested communicative goals. On the paralinguistic scale – comprehension, the subject obtains 1 point if he understands the type of communicative act or the correct emotion expressed; in production tasks, the subject scores 1 mark if he produces a communicative act using the appropriate paralinguistic indicators, adequate with respect to the type of communicative act that has been proposed. On the context scale – comprehension, the subject obtains 1 point if he recognizes that there is something inadequate in the proposed communicative exchange with respect to the context/situation, or to the rules underlying good communicative exchanges; on production tasks, the subject gets 1 mark if he produces a communicative act appropriate to the context or the situation, with respect to the formality or informality required.

For a detailed description of the scoring criteria, see Angeleri et al. (2012) and Bosco et al. (2012a). The psychometric properties of the ABaCo are reported in Sacco et al. (2008): all scales had satisfactory to excellent internal consistency, and the ABaCo demonstrated excellent inter-rater agreement. The neuropsychological and ToM tests were also scored according to the relevant criteria described in the literature for each test.

## Results

### Communicative – Pragmatic Assessment

We conducted a paired-samples *t*-test analysis to verify the effectiveness of the rehabilitative program, and analyze trends in patients’ performance on the equivalent forms of the ABaCo in the three assessment phases.

**FIGURE 2:**
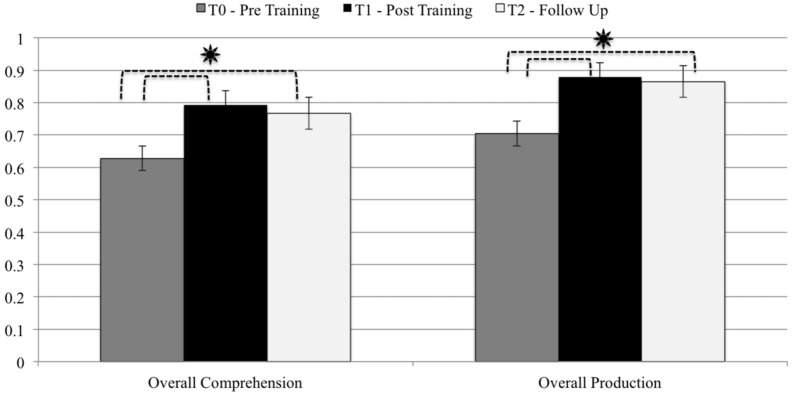
**Comparison between the average scores obtained on production and comprehension tasks, considered overall, at T0 – Pre-Training, T1 – Post-Training, and T2 - Follow-Up.** ^∗^*p* < 0.01.

First, we investigated whether the patients’ sub-diagnoses (different types of schizophrenia according to the DSM IV classification) could have influenced their communicative performance on the ABaCo scales: our analysis revealed no effect from belonging to a particular subgroup (Kruskal–Wallis test: 0.141 < *H*_(2)_ < 4.997; 0.082 < *p* < 0.932). We therefore considered the group as a whole. Considering the patients’ communicative abilities overall, we observed a significant improvement in performance at T1 (post-training) compared to that measured at T0 (pre-training) on both comprehension (*t*-test; *t* = 5.239; *p* < 0.0001) and production tasks (*t* = 4.143; *p* = 0.001). These improvements were stable even 3 months after completing the treatment, as shown by the comparison between scores obtained at T0 (pre-training) and at the Follow-Up assessment, on both comprehension (*t* = 4.039; *p* = 0.001) and production tasks (*t* = 4.040; *p* = 0.001; see **Figure 2**).

**FIGURE 3:**
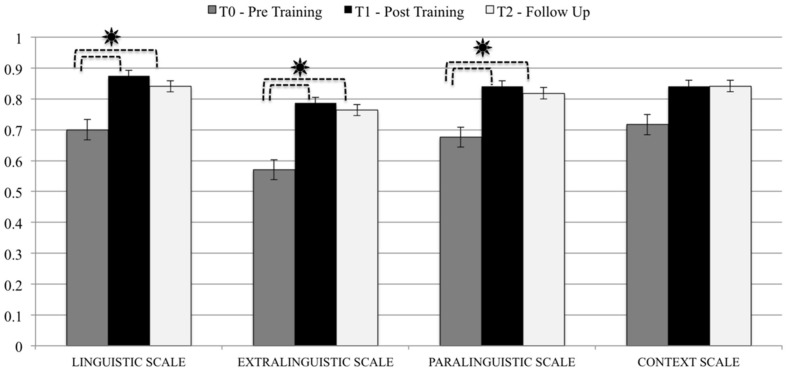
**Comparison between the average scores obtained on the ABaCo scales T0 – Pre-Training, T1 – Post-Training, and T2 – Follow-Up.** ^∗^*p* < 0.01.

In particular, we noted significant improvements on almost all of the ABaCo scales (considering comprehension and production together), namely on the Linguistic (*t* = 3.817; *p* = 0.002), Extra-Linguistic (*t* = 5.138; *p* < 0.0001) and Paralinguistic Scales (*t* = 3.152; *p* = 0.006); the improvements on the Context Scale were at the limit of statistical significance (*t* = 2.063; *p* = 0.056). The improvements were stable across all scales even after 3 months from the end of the remediation program, as shown by the comparison between scores on the Linguistic, Extralinguistic and Paralinguistic Scales, obtained at T0 (pre-training) and at the Follow-Up assessment (3.908 < *t* < 4.869; 0.0001 < *p* < 0.002). The comparison of the scores obtained at T1 and Follow-Up on the Context Scale were, again, only close to statistical significance (*t* = 1.871; *p* = 0.08; see **Figure 3**).

### Cognitive and Theory of Mind Assessment

At T0 and T1 we also administered a series of neuropsychological and ToM tests. The analysis of these did not reveal any statistically significant difference between pre- and post-training performance: Attentive Matrices (*t*-test: *t* = 0.048; *p* = 0.96), Trial Making test (*t* = 0.343; *p* = 0.74), Verbal Span (*t* = 0.111; *p* = 0.91), Spatial Span (*t* = 0.414; *p* = 0.685), Immediate and Deferred Recall test for long-term verbal memory task (*t* = 1.0; *p* = 0.33), Tower of London (*t* = 1.79; *p* = 0.09), Raven’s Standard Progressive Matrices (*t* = 1.62; *p* = 0.13), Modified Card Sorting test – Nelson (*t* = 1.49; *p* = 0.16), Sally and Ann task (*t* = 0.56; *p* = 0.58), Strange Stories task (*t* = 1.52; *p* = 0.15). The only exception was a significant improvement on the Aachener Aphasic test – Denomination Scale (AAT; *t* = 2.74; *p* = 0.02; see **Table 4**).

**Table 4:** Average scores obtained on the neuropsychological and ToM tests at T0 – Pre-Training and T1 – Post-Training.

	T0 – Pre-Training %	T1 – Post-Training %
**Neuropsychological and ToM tests**		
Attentive Matrices	65.24	65.12
Trial Making test	63.33	66.67
Verbal Span	35.33	35.0
Spatial Span	49.33	50.67
Immediate and Deferred Recall	57.39	63.19
Tower of London	61.20	65.65
SPM Raven	44.1	56.67
Modified Card Sorting test	79.44	82.78
Sally and Ann task	78.57	85.71
Strange Stories task	54.44	66.67
AAT – Denomination scale	95.39	97.72

## Discussion

The aim of the present research was to verify the effectiveness of a new remediation program, CPT, in improving communicative-pragmatic performance in a sample of individuals with schizophrenia. The program’s efficacy was measured by administering, before and after training, the equivalent forms of the Assessment Battery for Communication (Bosco et al., 2012a), a tool that is able to provide a complete overview of the communicative abilities of these patients, taking into account a wide range of pragmatic phenomena such as, for example, direct and indirect speech acts, irony and deceit, expressed through different communication modalities, i.e., linguistic, extralinguistic, paralinguistic, in addition to social appropriateness and adequacy to the context in which a communicative act is proffered.

Using the equivalent forms of the same tool in different assessment phases reduces the possibility of the results being attributable to factors such as practice and memory. All the patients were tested at the beginning of the research program using form A of the ABaCo in order to verify the presence of communicative-pragmatic deficits, detected by comparing their performance with normative data for the ABaCo (Angeleri et al., 2012). Subsequently the patients attended the CPT program twice a week for a total of 10 weeks, under the guidance of a psychologist, after which they were tested using form B of the Battery.

The results of the post-treatment tests revealed a significant improvement in patients’ performance on comprehension and production tasks for all the scales of the ABaCo, with the sole exception of the context scale, which was only close to statistical significance. In particular, we observed a significant improvement in linguistic abilities, i.e., the use of language for communicative purposes, and extralinguistic competence, i.e., use of gestures, and body movements. Moreover, at the end of the training program, the patients showed improved paralinguistic abilities, thus demonstrating a more fluent and appropriate use of tone of voice, gaze, and facial expressions. As regards the context scale, for which the difference in the results of the pre- and post-treatment assessments was only close to significance, we noted that this scale has fewer items than the others, which means it is probably less reliable and effective in detecting improvements in performance (see Bosco et al., 2012a). Our results also indicated that the improvement in communicative-pragmatic abilities remained stable over time: the effect of the treatment was still apparent at the follow-up assessment, thus demonstrating the continued efficacy of our program 3 months after the end of the treatment.

In addition to the equivalent forms of the ABaCo, a neuropsychological and ToM test battery was administered to the patients before and after the rehabilitation program. The results showed no significant differences in their performance pre- and post-treatment, with the exception of that on the Denomination scale of the AAT (Huber et al., 1983). This result is not surprising since the Denomination Scale of the AAT tests a person’s ability to correctly name an object and during the training program the patients performed exercises to improve this skill, for example, role playing activities in which they had to name objects correctly. Considered as a whole, these results testify a specific improvement in the ability on which the training program is focused, namely communicative-pragmatic ability.

One limitation of the present study is the absence of a control group. However, no significant improvements in other cognitive abilities, such as working memory, attention, planning, cognitive flexibility, or ToM were detected. Our rehabilitation program does not target these cognitive abilities, even though they do play a role in sustaining communicative ability (see Bibby and McDonald, 2005). The lack of significance in the improvement of such cognitive abilities tested before and after the treatment seems to suggest that the improvement observed in patients’ communicative-pragmatic performance is a specific result of our training.

Concerning the exception represented by the improvement on the Denomination scale of the AAT, this is a neuropsychological test that measures the patient’s ability to produce names of objects. The principal aim of the CPT program is not to improve patients’ ability to produce specific words. However, patients are trained to use language for communicative purposes and so the improvement in a specific component, i.e., word production, was not surprising as it is part of their linguistic communicative ability.

The CPT program primarily focused on the ability to manage the inferential chain in order to fill the gap that often exists between the literal utterance and the intended meaning, as in the case of indirect speech acts, deceitful, and ironic statements. In such communicative phenomena the comprehension of the literal meaning of the utterance is only the starting point in order to understand the speaker’s intended meaning (which does not simply correspond to the literally expressed one). The activities proposed during the treatment program were developed to make patients aware of the existence of such inferential processes and to encourage them, with the help of the therapist, to go beyond the literal meaning of a communicative act, expressed using either the linguistic or extralinguistic modality. During the Cognitive-Pragmatic Treatment program, for instance, the therapist pointed out that interpreting the literal utterance is not necessarily the same as understanding the communicative intention, and that it is important to consider any possible alternative meanings with respect to what the speaker actually says in order to comprehend exactly what he or she intended to communicate. The therapist also encouraged patients to consider all the expressive modalities (linguistic, extralinguistic and paralinguistic) that could help them to understand the meaning conveyed by the speaker, and to bear in mind the context in which the communicative act was proffered. By considering this perspective, the CPT program differs from other social skills training programs (for a review see Kurtz and Mueser, 2008), since its goal is not to teach patients how to behave in specific everyday life situations. Taken as a whole, these preliminary results are in line with previous research demonstrating the efficacy of rehabilitation programs which address various aspects of the social-cognitive problems characteristic of patients with schizophrenia. In a recent meta-analysis, Kurtz and Richardson (2012) reviewed the available literature on interventions to improve social skills of patients with schizophrenia, i.e., treatment focused on improving patients’ abilities to understand, perceive, and interpret the social context. Their review focuses on the main domain of social cognition, that is: facial affect recognition, perception of social cues, such as body language or voice intonation, ToM, and attributional style, i.e., the capacity to correctly attribute the causes of events. The authors indicated moderate-to-large effects of social-cognitive training procedures on facial expression recognition and small-to-moderate effects of such training programs on ToM abilities. In a review on social skills, Kurtz and Mueser (2008) reported that the average effect size of these interventions on psychosocial functioning is highly significant and consistent across studies, supporting the utility of social skills training in improving functional outcomes in these individuals, such as social adjustment and independent living. The role of rehabilitation programs in the treatment of schizophrenia is particularly relevant considering that antipsychotic medications have a limited effect in schizophrenia on cognitive functions, as for example attention, working memory, reasoning, and problem solving (Marder, 2006) and, regardless of the efficacy of antipsychotic medication in reducing psychotic symptoms, patients with schizophrenia are often severely impaired in the domains of communication, interpersonal interaction and social functioning (Kayser et al., 2006).

## Conclusion

Our preliminary findings seem to support the effectiveness of the CPT program in improving and enhancing communicative-pragmatic abilities in individuals with schizophrenia. However, also in view of the small number of participants in our study, further research is still necessary to generalize the results to the population of patients suffering from such pathology. Furthermore, future studies should include a control group of participants. Nonetheless, to the best of our knowledge, this is the first program specifically created to overcome communicative-pragmatic difficulties and to be administrated to a group of patients with schizophrenia.

Though only preliminary, these results also appear to be important in view of the fact that antipsychotic medications have a limited effect in schizophrenia on cognitive functions, as for example attention, working memory, reasoning, and problem solving (Marder, 2006). Communicative abilities allow people to relate to one another: an impairment in this domain may be responsible for unsatisfactory social interactions and improvement in communicative skills may increase patients’ quality of life. Schizophrenia is a complex pathology and different treatments, specifically focusing on social cognitive (Penn et al., 2007), metacognitive (Roncone et al., 2004; Moritz et al., 2005, 2010), and affective (Frommann et al., 2003; Wölwer et al., 2005) aspects have been developed in order to reduce patients’ symptomatology. However, at present and to our knowledge, no treatment specifically focuses on improving the communicative-pragmatic impairment exhibited by patients with schizophrenia. The CPT is the first attempt in this direction and could be considered as complementary to existing programs.

Unlike other rehabilitation programs, such as social skills training, our treatment specifically targets pragmatic abilities, which have been shown to be impaired in schizophrenia. We thus suggest that CPT training could be useful if administered in addition to other cognitive treatments already described in the relevant literature.

## Author Contributions

FB has supervised the whole project, both for what concerns the administration of the rehabilitative sessions and the preparation of the paper. IG conducted the rehabilitative sessions and was responsible for the assessment procedures pre and post-training. She took care of the methods and results sections of the paper. LG was responsible for the recruitment of the patients, the diagnosis and the patients’ symptomatology descriptions of the patients (e.g., PANSS). KS took care of the neuropsychological assessment and supervised the statistical analysis.

## Conflict of Interest Statement

The authors declare that the research was conducted in the absence of any commercial or financial relationships that could be construed as a potential conflict of interest. The reviewer MK and handling Editor declared their shared affiliation, and the handling Editor states that the process nevertheless met the standards of a fair and objective review.
