# Supplementary material for: Communicative-Pragmatic Treatment in Schizophrenia: A Pilot Study
Source: Front Psychol. 2016 Feb 23;7:166. doi: 10.3389/fpsyg.2016.00166 (PMC4762993; doi:10.3389/fpsyg.2016.00166)
Supplement: Supplementary file 1 [file Data_Sheet_1.DOCX]

**Appendix A**

**Structure of a typical rehabilitative session with examples taken from**

**Session 6 – Extra-linguistic ability**

*- Introduction and explanation of the content of the on-going session:* explicit reference to daily life situations where the topic of the session plays an important role. Brief summary of what has been done in the previous sessions.

- *Comprehension activities:* Video-taped scenes have been specifically created for the purpose of the training program. In each scene, two actors interact using the specific communication modality on which the session is based (i.e. mainly through language in linguistic sessions, mainly through gestures in the extra-linguistic session and so on). Three different video-taped scenes are shown to the patients during each session:

(1) a film clip where two actors play out a real-life situation implying complex communicative interchanges, such as ironic or deceitful statements. An example is:

*Scene*: Roberta is dusting the bookcase in her living room. While she is moving books to clean better, a whole row of books falls down on the floor. Luca, who was in the living room, focused on his own activities, look ironically at Roberta and makes a gesture with his hand as if to say "Great job!"

(2) a film clip showing a communication failure, where the actors do not achieve their communicative intents. An example is:

*Scene:* Luna is chatting with a friend on the phone. Her father passes close to her and he throws her daughter a glance of reproach, glancing at the clock. Misunderstanding, Luna looks at her own watch and nods his father with her hand that it’s half past four.

(3) a film clip showing the same situation as above, but where the actors’ understand their communicative intents and the communicative interaction is successful. An example is:

*Scene:* Luna is chatting with a friend on the phone. Her father passes close to her and he throws her daughter a glance of reproach, glancing at the clock. Then he passes close a second time and he makes a brusque sign pointing to the clock. Luna nods his father with her hand “Just a minute more”.

*Discussion* on the interaction depicted in the scenes in order to stimulate the patients’ comprehension of the situations presented. (What happened? Was the girl’s behavior appropriate to the situation and to the request? Why? What could she have said?). The discussion is also aimed at improving their discourse coherence and facilitating the participants to interact with each other and to introduce compensatory communication strategies.

*- Production activities*: Role playing activities devised for the specific purpose of the training program, aimed to offer the patients the possibility of practicing their communication strategies and get feedback in a protected setting. Patients are invited to conduct in-group conversations, in order to stimulate their ability to use contextual elements, as proposed by the theory of referential communication.

An example is:

Mr X: You invited a friend of yours at your place for lunch. You really would like your friend to help you in setting the table and cooking. Indeed, you know well your friend is kind a slacker and you are definitely annoyed by his behavior.

Mr Y: You're a guest at your friend's house. As usual, you want to find some good excuses not to set the table and do not help with cooking. As always, you hope to be able to enjoy a dinner without effort, thus you are ready to provide good excuses.

Moreover, specific sessions are devoted to enhancing various aspects of communication, such as the ability to recognize and correctly use facial expressions and prosody.

*Discussion* on the role play observed.

*Conclusion and homework,* in order to give patients the possibility to practice and reinforce the aspects of communication addressed during the session.
